# Spatial ligand–receptor gene annotations carry conditional heritability association across human tissues

**DOI:** 10.64898/2026.03.29.715138

**Published:** 2026-08-04

**Authors:** Chen Yang, Xianyang Zhang, Jun Chen

## Abstract

Methods that map genetic risk to cells do not directly test whether spatially organized ligand–receptor (LR) gene annotations carry conditional heritability association. Here we introduce EdgeMap, which scores each gene by the spatial activity of its LR contexts in spatial transcriptomics data, maps these scores alongside cell-intrinsic annotations to SNP-level LD scores, and tests both jointly against GWAS summary statistics. Across 17 traits and five human tissues, edge ***z*** -scores are systematically higher in biologically matched tissues: all 15 traits with both matched and unmatched tissue classes show this ordering (median **Δ *z* = 1.51** ; sign ***P* = 3.1 × 10 ^−5^** ), and 11 of 13 nominal-positive associations concentrate in the biologically matched set ( ***P* = 1 × 10 ^−4^** ). The pattern survives broad LR-gene-class and cell-type composition controls. Donor-level cross-section summaries, independent GWAS replication for LDL and CAD, and cell-segmented Visium HD liver data provide convergent support. A secondary analysis prioritizes constituent genes within active LR contexts; sixteen of 31 prioritized genes are absent from standard gene-level methods. These results establish spatially weighted LR-gene annotations as a complementary layer for interpreting complex-trait genetic architecture.

## Full Text Availability

The license terms selected by the author(s) for this preprint version do not permit archiving in PMC. The full text is available from the preprint server.

